# General Practitioner’s Experience of Public-Private Partnerships to Develop Team-Based Care: A Qualitative Study

**DOI:** 10.3389/ijph.2023.1606453

**Published:** 2023-11-14

**Authors:** Muriel Schütz Leuthold, Fatima El-Hakmaoui, Nicolas Senn, Christine Cohidon

**Affiliations:** Department of Family Medicine, Center for Primary Care and Public Health (Unisanté), University of Lausanne, Lausanne, Switzerland

**Keywords:** general practitioner, reform of the PHC system, multidisciplinary health team, family medicine, public-private partnership

## Abstract

**Objectives:** A tripartite public–private partnership was established between GPs’ practices, public health authorities and a university department of family medicine, to develop multidisciplinary teams and integrate nurses into GPs’ practices. The present paper describes the points of view of the GPs involved in this collaboration.

**Methods:** We conducted a qualitative study, with data coming from eight interviews with GPs, one from each practice. We also used the facilitator’s project diary to complete the discussion.

**Results:** The principal issue discussed was the financial aspects of the collaboration. GPs are generally satisfied, but time spent coordinating with nurses and transferring activities made them fear financial losses. Secondly, the partnership with public health authorities was well appreciated, but not clear enough. Some aspects of the partnership, such as referring patient to the nurse should have been better defined et controlled. The last aspect was the academic support. It allowed reducing GPs’ workload in training nurses and supporting the project implementation within the GPs’ practice.

**Conclusion:** GPs have a positive point of view of such public-private partnership and saw an opportunity to be involved in developing public health policies.

## Introduction

According to Jabbarpour, “*Team-based care is the cornerstone of practice transformation*” since “*evidence suggests that a team-based structure is essential if our primary care workforce is to meet the chronic and preventive care needs of our population*” [1]. Indeed, the development of multidisciplinary primary care (PC) teams seems to be one of the most appropriate solutions with which to address the challenges facing today’s healthcare systems, offering many advantages regarding the coordination, development, and reinforcement of new activities (prevention), and providing holistic care, shared workloads, and fewer unnecessary care interventions [2–4]. Indeed, although many Western countries, especially in North America, have started transforming their PC systems in this direction, progress has been different in Europe [5–12]. In countries like Switzerland, the PC system, via GPs’ practices, has become increasingly overwhelmed, although it is still able to meet most of the population’s needs in most parts of the country in a timely manner [13]. In this context, transforming the country’s PC model does not seem to be a necessity for many healthcare professionals, but for others, this transformation must occur by increasing the number of GPs trained in Switzerland [14, 15]. Some isolated initiatives have transformed GP-centered PC models into interprofessional teams [16–19]. Some of them show promising results by integrating, for example, advanced practice nurses [16, 17, 20] or specialized nurses [18]. However, they often cannot be deployed at a large scale because no adequate legal framework and funding mechanisms exist to support them [16–18, 20–22]. This is also the case in numerous other European countries. Indeed, in countries where GP-centered PC models are funded using fee-for-service systems, it is often difficult to develop team-based care because those systems will not pay for the services provided by nurses or social workers [23]. Despite of the obvious synergies and interactions between public health and primary care regarding the population’s health, safety, health surveillance, and planning [24], closer collaboration with the public health authorities should be a good way to transform PC systems, especially to develop interprofessional PC teams [25].

In Switzerland, most PC practices are privately owned and are operated by GPs alone or teams of GPs (family physicians) and medical assistants. GPs are paid for the types of medical acts they perform and the lengths of time these take—a fee- and time-for-services basis. This includes a part funding their medical assistants, who are GPs’ employees [26]. In 2015, regional public health authorities mandated an academic department of family medicine (DFM) to develop a project to improve PC coordination and, more generally, create new, interprofessional, PC teams. Based on the literature and local experts’ opinions, DFM proposed a new PC model made up of four components. The main component is the development of interprofessional PC teams that integrate a nurse into general practices. The other three components are valuable tools for nurses, i.e., care plans for chronically ill patients, the use of electronic patient health records, and patient empanelment list systems to develop collective activities such as health promotion and prevention [21]. After this important conceptual step, in 2019, we launched a regional pilot project. For this purpose, a particular public-private partnership including public health authorities, private general practices and the DMF was set up. Contrary to the usual partnerships, the investment came from the public entity. The canton’s public health authorities supported the project by financing nine nurses’ salaries into eight GPs’ practices, and they also enabled the recruitment of a facilitator to implement this new PC model and provided the resources needed to evaluate the project. The public health authorities hope to progressively expand the number of practices adopting the model and generalize it sustainably throughout the canton.

The present article aims to present the experiences of the GPs involved in the project, including the pros and cons of this public–private partnership.

## Methods

We performed a qualitative analysis covering the eight general practices involved in the project in 2021–22. We used the Consolidated Criteria for Reporting Qualitative Research (COREQ), a 32-item checklist for conducting and reporting this type of study [27].

### Population

Eight general medical practices participated in the pilot project, covering both urban and rural areas. These practices employed nine nurses (5.86 full-time equivalents, FTEs), 30–35 medical assistants (MAs, 25.95–30 FTEs), and 24 GPs (19.6 FTEs). This region is Switzerland’s most populous French-speaking area, with approximately 800,000 inhabitants and 900 GPs working in either solo or group private practices.

Inclusion in the pilot project was voluntary for the GPs’ practices and for the GPs working in them. Participation was officialized by tripartite contracts signed between GPs’ practices, public health authorities, and an academic department of family medicine. Contracts described the terms of the collaboration: health authorities guaranteed nurses’ salaries and operating costs; the DFM provided administrative support (e.g., human resources), help with project implementation (including evaluation) through the facilitator, and training for nurses and GPs’ practices teams; and GPs agreeing to participate in the project undertook to comply with the nurses’ description in order to allow the nursing activities’ implementation and to take part in its evaluation. Contracts defining the collaboration and nurses’ job descriptions were developed with GPs, and these contracts were relatively flexible so that they could be adapted to each practice’s particular context.

### Data Collection

Our main sources of qualitative data were the semi-structured interviews conducted with eight GPs involved in the pilot project—one from each practice. These occurred at the end of the pilot project, between March and July 2023. Each interview lasted 60–90 min and was designed to collect information about the new interprofessional organization’s functioning and acceptability, GPs’ satisfaction regarding the partnership between their practices and the health authorities, the support received by the academic team involved in the project, and the training provided to the nurses. All the interviews were audio recorded, and all personal data and references were removed from them. To enrich the discussion, we also used data from the facilitator’s project diary.

### Data Analysis

The audio recordings of the eight interviews were transcribed in full, and data were imported and coded using MAXQDA Analytics pro 2022 software. We carried out a thematic analysis of the texts using an inductive approach to extract categories, assemble these into themes, and then formulate hypotheses [28, 29]. Results were regularly discussed within the research team in order to identify the main categories and themes emerging.

## Results

Several categories and themes regarding the tripartite partnership emerged from the interviews. The main issues mentioned by the GPs were the financial aspects of the partnership and collaboration with the public health authorities and the DFM.

### Financial Aspects of the Partnership

GPs viewed a number of different aspects of the partnership framework to be important. One major issue was the financial constraints perceived by GPs. In general, the GPs viewed the funding provided by the health authorities as a facilitator. It allowed nurses to develop new activities and services without putting financial pressure on the practices. Billing the care delivered by nurses would have been a barrier to the project’s implementation, not only in terms of its general development but also regarding patient acceptance. Billing nursing services could also have discouraged patients from consulting them, as one GP noted: “*She cannot work for free*, *but that has also really been a big factor in getting* [*patients*] *to agree to be seen by the nurse.*”

The funding provided was also sufficient to convince GPs to participate in the project without fear of financial loss to the practice. The amount paid to the practices by public health authorities covered the nurses’ salaries, part of the costs of using a consultation room, and additional compensation for the workload caused by project evaluation. GPs found it appropriate that this amount also covered the cost of the nurse’s office space. The financial model and the partnership should continue with the payment of the nursing salary and the costs of the nurse’s office space, as one GP said: “*The model should continue to work in the way it’s been set up*, *in terms of financing. […] The conditions should stay as they are […]*.”

However, despite the extra funding, some GPs felt that their practices were losing out financially. Financed on a fee- and time-for-services basis, they felt that they spent a lot of non-invoiceable time learning how to work with nurses and training them on how to practice PC in the context of GP’s practices. Some GPs nevertheless believed that this loss would be made up later thanks to the healthcare provided by the nurses to the practice’s patients: “*It was not easy at first, because […] it’s time spent [with the nurse] that we cannot bill*. *[…] We bear the cost for this time [at first]*, *but in the end, there is a huge return on investment*.” However, the billing system does not encourage GPs to delegate more complex cases to nurses.

Depending on the activities nurses carry out, e.g., medical acts performed by the nurse instead of by the practice’s medical assistant, such as drug injections, some GPs thought their practice would suffer a financial loss, as one mentioned: “*If the medical assistant provides care*, *I get paid*, *which enables me to pay her*. *If my nursing colleague does it*, *I earn nothing*.” On the other hand, nurses taught medical assistants new skills and expanded their knowledge base: “*She had a beneficial impact because she brought the medical assistants knowledge and skills*, *[…] she could teach*, *and she gave some explanations on emergency care*. *She supported the medical assistants*.”

### Collaboration With Public Health Authorities

Overall, the collaborative tripartite model (public health authorities–research team—PC practice), especially their partnership with the public health authorities, was well-perceived by GPs. Within this collaborative effort, the public health authorities and the DFM showed their willingness to allow GPs’ practices some room for maneuver in the project’s implementation. Only a small number of the GPs found the model intrusive and rigid, as one stated: “*Other colleagues have seen this as government interference in our freedom [to practice]*. *For me*, *in the end*, *it’s the end result that interests me more [….]*. *Obviously*, *when it’s something to do with the government*, *things are always slower and always a little more rigid*.” Other GPs, however, declared that the contract and the partnership were too flexible. GPs were not obligated to refer patients to their practice nurses in order to allow them to develop new activities, such as follow-up of patients with care plan. Some GPs would have liked the public health authorities (via the DFM) to have better clarified and controlled this aspect of the partnership. The lack of clarity was detrimental to the implementation of nursing activities and also generated tensions within practices. As one GP remarked: “*My colleagues were involved in the project*, *but not really […] But perhaps [the DFM] could have helped to clarify things because the nurse had a half-empty consultation schedule, and then she was being paid for doing nothing*. *She felt uncomfortable*. *[…] it created quite a lot of tension between the GPs and the nurse*, *as well as between the GPs and the medical assistants*.”

The tripartite contract model, on the other hand, was much appreciated, especially the fact that the nurse was paid for and employed by the cantonal government. It removed any issues about nurses’ hierarchical relationships with GPs and thus encouraged collaborative relationships*.* “*It’s probably good because it removes the whole employer–employee issue*, *which would complicate the relationship […] it simplifies our collaboration*.” The nurses’ employment contracts, and the job description drawn up by the DFM, enabled nurses’ skills to be put to proper use, as one GP mentioned: “*Things are clear*: *she [the nurse] is a [DFM] employee with a job description from [the DFM]. But if she became a GPs’ practice employee*, *there is more temptations to do other […]*.” Hence, GPs were less likely to ask them to perform activities that were not mentioned in the job description. In terms of the job description’s content, some GPs considered it imprecise and unclear. For others, however, it needed no changes because it allowed for future adaptations: “*Maybe the job description should not be too rigid*, *which would enable it to be adapted to the reality*.”

Finally, for the GPs, these partnerships were also an opportunity to raise public health authorities’ awareness about patients’ common problems and needs and to influence future public health policy: “*It’s great! Maybe we’ll be able to be sentinels in the field*, *maybe public health authorities will learn that there may be critical situations in our population today*, *thanks to GPs’ practices*.”

### Collaboration With a University Department Dealing With Primary Care and Public Health

The DFM was responsible for the academic support that went into designing this new model of care and performing a scientific evaluation of the project. It was also involved in managing the project’s human resources—the nurses. Most of the GPs appreciated this support. However, two GPs’ practices encountered difficulties due to divergences in the management of those resources by [the DFM] and the GPs’ practices, as one GP mentioned: “*Sometimes it is complicated. There are, finally*, *two different HR [human resources] principles*.”

The DFM provided further support in the form of nursing training. The main training objectives were to facilitate the implementation of this new organizational model and encourage the development of nursing care activities based in GPs practices. This training lightened GPs’ workloads. Nevertheless, they would have preferred the training to have been given at the beginning of the pilot project. In their opinion, the content should also have been more focused on family medicine in order to improve nurses’ skills and autonomy. “*What was really lacking was the training […] and then everything to do with cardiovascular prevention*, *diabetes*.”

The final topic was the added value brought by the project’s facilitator. The facilitator was a member of the DFM’s research team, and her role was to help nurses implement the new organizational model within their new practices. She also played a key role with GPs during the project’s start-up phase by reinforcing messages to the teams regarding the project framework and accompanying the change process. As one GP said: “*She was a facilitator not only for the nurse but also for the team […] presenting and formalizing the project*.”

## Discussion

Our findings revealed that the participating GPs’ had generally responded positively to the public–private partnerships developed through the project. It was clear that for most of them, the financial support of the public health authorities was a prerequisite for embarking on such a profound change in the organizational model of PC. GPs in Switzerland are remunerated exclusively on a fee- and time-for-service basis, which enables them to pay their medical assistants and all their practice expenses. Current regulation makes it almost impossible to finance healthcare services by other professionals, such as nurses, in general practices. In another Swiss cantons, projects similar to this project was developed a few years ago, but despite its positive evaluation, one of them was stopped because the relevant public health authorities decided not to continue supporting it [18]. The three other projects integrating advanced practice nurses also encounter difficulties in their sustainability due to an unsuitable legal framework and financing mechanisms [16, 17, 19]. Examples from other countries, such as Canada or the United States, have shown that the organizational transformation of PC models, particularly building new PC teams, must go hand in hand with a transformation in the financing model [30, 31]. Traditional fee-for-service models are not appropriate when funding teams including physicians and the various other professionals who are not usually funded in this way. In most cases, blended payments have been introduced, including capitation, incentives, salary, and performance-related pay [25, 32].

In the present project, another public–private partnership appeared to be important in transforming the PC organizational model. This was the partnership with an academic institution working in the fields of public health and general medicine. We had not anticipated the importance of this component when the project commenced. This partnership was appreciated by all the PC professionals—both nurses and GPs—for several reasons. Firstly, the DFM is experienced and well-regarded institution in the fields of public health and primary care; collaboration with them was perceived as an asset by both GPs and nurses. Secondly, the administrative work of integrating the nurses into practice, i.e., contracts, job specifications, vacations, etc., was entirely managed by the DFM. GPs highlighted this as a key element in the project. The downside, for GPs, was that they had little control over nurses’ activities and schedules, but this seemed to be acceptable to them. Thirdly, because the project was also anchored within an academic context, all types of PC providers could benefit from the training provided. Indeed, it was easy for the DFM to provide nurses with training, helping them to take ownership of their roles. Finally, the support from the research team as a whole, particularly the project implementation facilitator, seemed to play a critical role in the transformation of the PC model. As the facilitator’s project diary confirmed, the facilitator helped to adapt the new model to GPs’ practices by involving GPs’ staff and accompanying them through the changes. Literature on the subject confirms the value of having a facilitator to smooth new care implementation processes in PC [33, 34].

For DFM researchers, as a PC health services research (HSR) team, conducting research on building sustainable models of PC transformation was very interesting and instructive. The opportunity to carry out real-world projects is an asset as HSR deals with complex interventions and processes. Being involved in a tripartite partnership and interacting directly with both general practices and public health authorities allowed us to evaluate the project using realistic approaches, which is particularly appropriate [22, 35, 36].

The present paper mainly reported GPs’ points of view regarding the project’s public–private partnership. Indeed, in a system involving privately run family medicine practices, this is often the limiting partner. However, the pilot project partnerships involve several partners, and each one must benefit from the arrangement. We mentioned above the benefits for an HSR team. Public health authorities can find really strong reasons for participating as well. Indeed, having public health professionals (or at least healthcare professionals funded by public health authorities) working in PC settings can enable better implementation of public health programs and relay public health policy messages. In addition, monitoring PC activities is another interesting advantage, in a context where data are generally lacking. Monitoring would be particularly interesting if public health authorities funded GPs’ private practices to implement new public health policies and wanted to monitor them. However, data collection in GPs’ practices faces several barriers. The diversity of types of electronic medical records and designs does not allow for easy data extraction in Switzerland. Furthermore, buy-in by GPs would be essential but perhaps difficult to obtain. Yet, in an extraordinary context, such as the COVID-19 pandemic, such public–private partnerships could reach their full potential. As in some Canadian provinces, the project nurses were particularly involved in PC support activities, such as triage, clinical management, team support, and organizational and administrative tasks [37, 38].

GPs see the obvious benefits of having local authorities fund a supplementary healthcare professional in their practice at hardly any cost to them. However, not all GPs will wish to join a public–private partnership of the type described here. In Switzerland, and probably in other countries such as France or Germany, some GPs are very attached to their private practice status. A partnership with public health authorities, or even simply with academic experts, is often perceived as a potential loss of autonomy and a loss of control over the quality of care they deliver. Moreover, any transformation in financing models, towards more mixed models, often gives rise to fears of a loss of income [39]. The challenge, therefore, is often convincing GPs of the benefits of transforming the organizational and financing sides of their practice. The positive experiences of this found in the literature, such as in Ontario, Canada [25], are helpful.

In the long term, the scalability and sustainability of such model will not depend only on convincing doctors in private practice to change their organization. The various stakeholders in this partnership have also to work to consolidate the legislative and financial framework. The current local political context is particularly favorable to the development and financing of such partnerships. However, the partnership remains fragile and could come to a halt if it loses political interest and support.

### Limitations

Our research project had some limitations. Due to funding, the sample size is relatively small with only eight GPs’ practices. The GPs’ practices involved in the pilot project had also all volunteered to participate. These GPs were particularly interested in working in interprofessional organizational settings with nurses. Indeed, three of the practices selected had employed nurses before the pilot project but benefited from the possibility of financing them through the project. Despite this, some GPs did not collaborate with the nurses by, for example, not referring patients to her.

The partnership also had some limitations. Only the DFM signed contracts with GPs’ practices; the public health authorities were not directly involved in this process, so the DFM was entirely responsible for ensuring compliance. In addition, the project had to be attractive to GPs; they could not lose out financially, and we wanted to avoid imposing too many constraints on them. Hence, GPs were not accountable to the public health authorities and, more broadly, to the population financing the nurses through their taxes. If such an organizational model had to be implemented at a large scale, the partnership would probably have to evolve so that the public health authorities were the guarantors of the proper use of resources.

### Conclusion

Beyond the project and the development of interprofessional PC teams, greater collaboration between private PC practices, public health authorities, and/or public health experts could create genuine opportunities to improve PC systems. These closer working relationships lead to partnerships and better knowledge and understanding of each other’s activities and missions. From the public health authorities’ perspective, this can help to generate more appropriate decisions and policies. For PC professionals, this could be synonymous with their integration and participation in public health decisions, policy orientations, and activities. This will become increasingly essential if PC providers are to meet the population’s growing needs for this type of care and ensure that it is sustainable in the future.
